# Evaluation of Antibacterial Efficacy of Photodynamic Therapy vs. 2.5% NaOCl against *E. faecalis*-infected Root Canals Using Real-time PCR Technique

**DOI:** 10.4317/jced.53526

**Published:** 2017-04-01

**Authors:** Maryam Janani, Farnaz Jafari, Mohammad Samiei, Farzaneh Lotfipour, Ailar Nakhlband, Negin Ghasemi, Tannaz Salari

**Affiliations:** 1 Assistant Professor, Endodontics Department, Dentistry Faculty, Tabriz University of Medical Sciences; 2 Department of Endodontics, Dental School, Tabriz Branch, Islamic Azad University; 3 Associate Professor, Endodontics Department, Dentistry Faculty, Tabriz University of Medical Sciences; 4 Professor, Faculty of Pharmacy, Tabriz University of Medical Sciences, Tabriz, Iran; 5 Research Center for Pharmaceutical nanotechnology, Tabriz University of Medical Sciences; 6 Dentist, Tabriz University of Medical Sciences

## Abstract

**Background:**

Bacteria like *E. faecalis* can produce intra- and extra-radicular biofilms. Theoretically, the adjustable penetration ability of lasers enables better access to root canal system. Therefore the aim of the present study was to compare the ability of photoactivated laser and 2.5% NaOCl irrigation solution to eliminate *E. faecalis* from the root canals by real-time PCR technique.

**Material and Methods:**

Sixty extracted human upper central incisors were selected and sterilized in an autoclave. The root canals were infected with *E. faecalis* (PTCC 1237, Persian Type Culture Collection, Iran) and then incubated for 24 hours. The samples were randomly divided into 3 groups. No intervention was made in the control group (group 1). In group 2, laser therapy was performed with a power of 100 mW by diode laser for 120 seconds. In group 3, the canals were irrigated with 5 mL of 2.5% NaOCl; then all the samples were sonicated in 15 mL of normal saline in test tubes in order to isolate the bacteria. DNA extraction was performed followed by real-time PCR technique for all the samples.

**Results:**

Inhibition of bacterial growth in all the experimental samples was significantly more than that in the control group. There was a significant difference between photodynamic therapy and 2.5% NaOCl. The effect of NaOCl in all the samples was better than photodynamic therapy. The results of the mean CT (cyclic threshold) were 40, 30.2 and 15.35 for 2.5% NaOCl, photodynamic therapy and control group, respectively.

**Conclusions:**

Based on the results of this experimental study, 2.5% NaOCl eliminated *E. faecalis* from infected root canals more effectively compared to photodynamic therapy.

** Key words:**Photoactivated laser, Enterococcus faecalis, antibacterial agents, sodium hypochlorite.

## Introduction

A large number of microorganisms, including *E. faecalis*, have an important role in the etiology of periradicular lesions after root canal treatment (1,2). *E. faecalis* has been isolated from 24-77% of periradicular lesions (2). This gram-positive facultative anaerobic microorganism is normally found in the oral cavity normal flora. It can penetrate into the dentinal tubules and cannot be completely eliminated during root canal preparation; therefore, it is one of etiologic factors for the failure of endodontic treatments (3,4).

Laser beams can be used as an adjunct in endodontic treatment (5). It has been reported that dental lasers can improve access to areas of the tubular network that were previously inaccessible, an addition to the elimination of debris and removal of the smear layer (6). Therefore, they can decrease bacterial counts in the root canal (6).

A new technique for the elimination of microorganisms from the root canal system is the use of low-level laser beams (7). In this technique, light-sensitive materials such as tolonium chloride are used. These materials bind to the cell membrane of bacteria, making them sensitive to laser beams. Due to the irradiation of laser beams, the molecules of the light-sensitive material move to higher energy levels and then transfer this excess energy to oxygen molecules to create free oxygen radicals. These free radicals destroy bacterial proteins, nucleic acid and lipids. This technique is referred to as photoactivated disinfection (PAD) (8). One of the advantages of this technique is an increase in heat <0.5°C, which is not significant clinically. PAD, in addition to the elimination of bacteria, accelerates bone formation processes in the periradicular area and is a strong stimulus for the healing of bone (7). This technique does not injure the host tissues and is very effective in eliminating *E. faecalis* from the root canal space (9).

Different techniques have been used for the evaluation of bacteria in the oral cavity and root canal system (10,11), including bacterial culture and colony counting technique as one of the oldest techniques. However, the limitations of this technique include the uncultivable nature of some microorganisms, the high technique sensitivity during sampling and transfer to the laboratory, dependence on the technician’s skill and expertise, etc (10). Molecular techniques have been introduced for identification of microorganisms with various advantages, including detection of cultivable and uncultivable species, high accuracy and specificity, high sensitivity, no need for controlling anaerobic conditions, the capacity to carry it out during antimicrobial therapy, easy transfer of the samples to the laboratory and detection of non-viable microorganisms, this last case is considered an advantage and a disadvantage. Therefore, this technique has become very popular (10). Various studies have used PCR-based techniques to evaluate endodontic and root canal microorganisms (12-14). Studies have shown that the accuracy of this new technique is several times that of culturing and colony counting technique (15).

Since no studies to date have used real-time PCR in this respect, this study was designed to compare the capacity of photoactivated laser technique in eliminating *E. faecalis* from the root canal system in comparison to irrigation with 2.5% NaOCl solution.

## Material and Methods

-Preparation of Samples

First, 60 maxillary central incisors with mature apices and straight roots, without any anatomic anomalies, were selected. The teeth had been extracted for periodontal reasons. The teeth were stored in 3% chloramines T solution until used for the purpose of the study. The root surfaces were cleaned with ultrasonic devices, and cracked teeth and those exhibiting calcification on radiographic views were excluded from the study. The tooth crowns were removed at CEJ with a disk (D&Z, Diamond, Germany) to leave a root length of approximately 12 mm. Then the working length was determined 1 mm short of the apical foramen with the use of a #20 K-Flexofile (Maillefer, Dentsply, Switzerland). Then the root canals were prepared with #4 and #3 Gates-Glidden drills (Maillefer, Dentsply, Switzerland) and #40, 10%, #35, 8% and 30, 6% RaCe rotary files (FKG, Switzerland) using the crown-down technique in a uniform manner. Normal saline solution was used to irrigate the root canals. The smear layer was removed with 5.25% NaOCl (Taj Corp, Tehran, Iran) for 3 minutes, followed by 17% EDTA (Pulpdent Corp, MA, USA) for another 3 minutes. The teeth were sterilized in an autoclave at 121°C under 15 psi for 20 minutes. To confirm sterilization of the teeth, they were incubated in brain-heart infusion broth (Merck, Darmstadt, Germany) at 37°C for 24 hours. Then a pure bacterial culture of *E. faecalis* (PTCC 1237, Persian type culture collection, Iran) gram-positive cocci was prepared. Before initiating the study, frozen bacteria (-20°C) were defrosted and incubated on brain-heart infusion broth agar medium (Merck, Darmstadt, Germany) enriched with 7% sheep blood at 37°C under aerobic conditions. Some colonies were retrieved from the plates and transferred to bile esculin sodium azide agar (Merck, Darmstadt, Germany) and once again cultured at 37°C for 24 hours under aerobic conditions.

The colonies growing on the surface of the agar medium were collected and adjusted to 0.5 standard McFarland concentration (2.5×108 CFU/mL) in normal saline solution with the use of spectrophotometry. Then 200 mL of the bacterial solution were transferred into the root canal lumens with a micropipette. After 48 hours, all the root canals were dried with sterile paper points. Then the samples were randomly assigned to 3 groups (n=20): group 1 (control); group 2, photoactivated laser group; and group 3, 2.5% NaOCl group. No intervention was carried out in the control group. In group 2, after placing 1.2 mg/L of tolonium chloride (PACT® Fluid Endo, Germany) for 3 seconds, the root canals underwent laser irradiation with the use of a flexible Denfotex endotip (Technologies Ltd), measuring 15 mm in length and 300 µm in diameter for 120 seconds. Diode laser (BWTEK Inc., Newark, DE), beams were used at a wavelength of 635 nm at an output power of 100 mW (16). The teeth in group 3 were irrigated with 5 mL of 2.5% NaOCl (Taj Corp, Tehran, Iran).

All the teeth were transferred into 15-mL test tubes containing normal saline solution and sonicated using a 34-kHz and 180-W sonicator (Modstar Sonic 1835 Italy) for 6 minutes to separate the bacteria attached to the canal walls and suspend them in normal saline solution.

-Extraction of DNA

DNA was extracted from the prepared samples and then evaluated with real-time PCR. The tooth samples were stored in a freezer at -20°C until the DNA was extracted. Under liquid nitrogen, 50 mg from each sample was converted into powder and a lysing buffer containing 10% SDS (NaCl, 150 mM; Tris, 15 mM; EDTA, 10 mM; pH=7.5) was added and incubated overnight in proteinase K at 60°C. After incorporation of 6-M NaOCl and centrifugation, the supernatant was separated and rinsing was carried out with cold ethanol. Then ethanol was eliminated and the DNA was purified in 100 mL of TE buffer (Tris, 10 mM; EDTA, 1 mM, pH=7-8) and stored at -20°C in a freezer. The concentration and quality of DNA were determined by a biophotometer at 260 and 280 nm wavelengths.

-Electrophoresis with agarose gel

First, 1% agarose gel was prepared by dissolving 1 g of agarose powder in 100 mL of buffer (Tris-Borate-EDTA 0.5X) and mixing with etidium bromide. Then the proliferation products of each gene were loaded in gel agar in the real-time PCR reaction and electrophoresis was carried out. This stage was carried out in order to confirm proliferation of the specific fragments of each gene and absence of non-specific products and pairing of the primers (dimer primer). In the present study, primers in which were prepared for previous studies were used.

-Enterococcus faecalis

The primers consisted of hflF:5-GCCAGATGGTTTACAAGCAC and hflR:5-TATTCCGTTTCTTCG and the target gene was GroES/El Chaperone protein.

-Thermal cycles 

The thermal cycles consisted of 32 cycles at 95°C for 25 seconds, 58°C for 40 seconds and 72°C for 90 seconds. To make sure of the absence of homology and complementation of the sequence of primers, their sequences in the BLAST website (NCBI) (www.ncbi.nlm.nih.gov/blast) were compared with nucleotide sequences in the other sections of the genome.

-Real-time PCR

In the present study, real-time PCR unit, model BIORAD IQ5 (Australia), was used. The thermal timetable of the machine was carried out in 3 stages and activation of DNA in the first stage, which results in denaturing of the molecules of polymerase, took place at 95°C for 10 minutes; the second stage occurred at 95°C for 15 seconds and 55°C for 1 minute for 42 consecutive cycles, and the final stage or the melting cure to draw the dissociation curve after the final stage of the reaction was selected at 60-95°C; the thermal changes were applied at a proper gradient. The reaction took place at a final volume of 25 µL in triplicate. The mixture of each reaction consisted of 5 µL of SYBR-Green PRC Master Mix (ABI Company, Germany) and forward-and-reverse primers, each at a volume of 1 µL with a concentration of 10 pM, genomic DNA (17) up to 20-100 ng/μL and distilled water were added to each to achieve a final volume of 25 µL. In the present study, a material with a green fluorescent dye was used, referred to as SYBR Green 1, Capable of being placed in a small groove of the two-stranded molecule to radiate a fluorescent light. The amount of the fluorescent light produced is directly related to the amount of the PRC product.

-Optimization of the Reaction

First, serial dilutions were prepared at 3, 6 and 9 pmol/µL in order to determine the proper concentration and function of primers. In addition, 25 and 50 ng of serial dilutions of DNA (control) were used in the real-time PCR tube for each gene in order to draw the standard curve. Based on the standard curve, the range of the optimal concentration of DNA pattern and the efficacy of the PCR was determined for the target gene. The reaction of serial dilutions for primers and standard DNA template took place for each gene in a dual manner in association with a non-template control (NTC) reaction. Changes in the target gene relative to the gene in question was evaluated and compared in the standard DNA of *E. faecalis*. Therefore, after completion of serial dilution reaction, from the standard DNA as a pattern, the standard curve was drawn for each gene based on the log of DNA concentration (the transverse axis) and the standard cycle or CT (the vertical axis). The standard curve gradient was used to calculate the efficiency of the proliferation reaction, which should be -3.0 to -3.7: PCR efficacy percentage = [10 (-1/slope)-1×100].

-Statistical Analysis

One-way ANOVA was used to compare the mean CT (cyclic threshold) between the three groups and if the test was significant, post hoc Tukey tests were used. Statistical significance was set at *P*≤0.05.

## Results

The results of post hoc Tukey tests revealed statistically significant differences in the mean CT between the positive control group and photodynamic and 2.5% NaOCl groups, with higher means of CT in both photodynamic therapy and 2.5% NaOCl groups compared to that in the positive control group (*P*<0.001). In addition, the results of Tukey test showed a significant difference in mean CT between the photodynamic therapy and 2.5% NaOCl groups, with higher mean CT in the 2.5% NaOCl group compared to the photodynamic therapy group (*P*<0.001). The means and standard deviations of CT in the positive control, photodynamic therapy and 25% NaOCl groups were 15.33±0.99, 30.20±2.29 and 40±0.0, respectively. In general, in the samples evaluated, the mean CT in 2.5% NaOCl group was higher than that in the three groups, and the mean CT in the positive control group was less than that in the other groups.

One-way ANOVA was used to compare the means of CT between the three groups under study. The results showed significant differences between the three groups (*P*<0.001). Post hoc Tukey tests were used to exactly determine the difference between each two group.  Table 1 presents the results of Tukey tests.

**Table 1:**
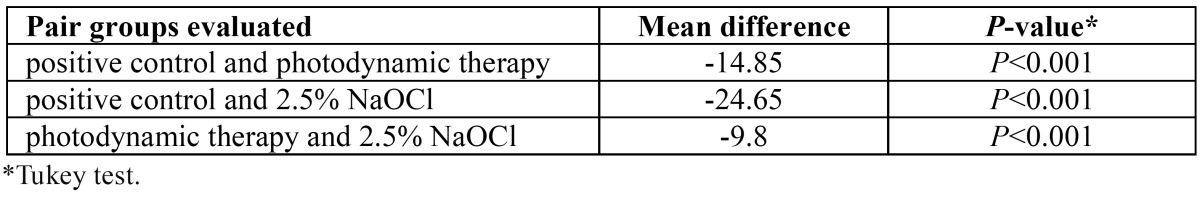
The results of post hoc Tukey tests.

## Discussion

*E. faecalis* in one of the etiologic factors for the failure of endodontic treatments (18,19). This microorganism can form intra- and extra-radicular biofilms, and it has been shown that these biofilms are very difficult to eliminate from the infected root canals (20). On the other hand, a large number of commonly used antibacterial agents might have no effect on the deep dentin layers (21). Different types of laser have so for been used in dentistry, especially in endodontics and the efficacy of many of them has been shown in eradication of *E. faecalis* (22,23). The advantage of lasers in this context is that their penetration depth can be controlled and as a result they can gain access to areas with complex anatomic structure (24).

NaOCl solution in used as an intracanal irrigation solution due to its antibacterial properties and its reaction with the organic compounds of the dental pulp such as amino acids and fatty acid to dissolve them. Its low viscosity allows it to easily penetrate into the root canal. Another advantage of NaOCl is its availability and low cost (25). Sodim hypochlorite solution also has some disadvantages like poor taste and high toxicity (26).

In the present study, the antibacterial effects of laser photodynamic therapy and 2.5% NaOCl were evaluated with real-time PCR technique in root canals contaminated with *E. faecalis*. Based on the results, both the antibacterial agents used in this study resulted in significant decreases in *E. faecalis* counts compared to the control group. In this context, there was a significant difference between the laser photodynamic therapy and NaOCl, with 2.5% NaOCl exhibiting higher efficacy than photodynamic therapy.

In relation to the application of Er,Cr:YSGG laser as an antibacterial agent with in the root canals, Eldeniz *et al.* evaluated 40 root canals contaminated with *E. faecalis* in 4 groups of control, irrigation with 3% NaOCl and two laser groups with 0.5-W laser beams based on the size of the apical foramen. The results showed significantly higher decreases in bacterial counts compared to the control group. In addition, such a decrease in bacterial counts in the 3% NaOCl group was more than that in the two laser groups (27).

In a study by Tennert *et al.* (28), the antibacterial effects of laser-activated NaOCl and laser alone on 72-hour-old *E. faecalis* biofilms, using the microbial culture techniques, were 92.7% and 99%, respectively.

Rios *et al.* (29) carried out an electron microscopic study on the effect of PDT + toluidine blue on *E. faecalis* and showed that root canals treated with PDT for 30 seconds exhibited only 2.9% of residual bacteria; however, in the group treated with a combination of PDT and 6% NaOCl, approximately 0.1% of bacteria remained after root canal therapy and this technique was considered an effective technique for decreasing microbial load of the root canal system, almost consistent with the results of the present study. However, in the present study, a lower concentration of NaOCl was used n order to decrease toxicity.

A study by Atieh (30) showed that real-time PCR was significantly more accurate in detecting P. gingivalis and A. actinomycetemcomitans compared to the culture technique. Zand *et al.* (11) carried out a study to determine colony forming units (CFUs) for the evaluation to the effect of photodynamic therapy and NaOCl on *E. faecalis* biofilms in different stages and concluded that photodynamic therapy and 2.5% NaOCl were effective in completely eliminating *E. faecalis* biofilms. However, in the present study, NaOCl was more effective than photodynamic therapy in eliminating bacteria, which might be attributed to the high sensitivity of real-time PCR technique in detecting bacteria. The difference between the results of the present study and above mentioned research can be justified with the results of Sedgley *et al.* (15). Another justification for the inconsistency of results may be more efficacy of sodium hypochlorite than laser in complete denaturation of bacteria which make it impossible to detect them by PCR.

Sedgley *et al.* (15) compared the real-time qPCR and the culture technique, in which salivary samples were collected from 30 subjects. The real-time qPCR and the culture technique were able to detect *E. faecalis* in 5 samples (17%) and 2 samples (7%), respectively. They concluded that real-time qPCR is more accurate than the culture technique.

Karale *et al.* (3) evaluated the effects of 3% NaOCl, 2% CHX, HFAC (high-frequency alternating current) and normal saline (control) on *E. faecalis*. The results showed that NaOCl, CHX and HFAC were effective in eliminating *E. faecalis* while NaOCl exhibited the highest antibacterial effect against *E. faecalis*, consistent with the results of the present study.

The results of studies by da Forta (31), Silva (32), Marinic (33) and Juric (34) in relation to the effect of photodynamic therapy compared to the positive control group on *E. faecalis* are consistent with the results of the present study; all the studies above have used microbial culture and colony count technique.

Like previous studies about antifungal activity of endodontic sealers (35), designing similar studies evaluating antifungal activity of laser against fungal infections of root canal system can be useful for future studies.

Since there are differences between different studies in relation to laser-activated materials, the techniques used for root canal cleaning and shaping, the type and power of the laser used, the concentration of NaOCl, the technique used to determine colony counts, the molecular technique use to detect bacteria and the age of the biofilms used, it is not possible to directly compare the results of the present study with those of other studies, and such differences justify the discrepancies between the results of different studies. The advantage of the present study was the application of sensitive molecular techniques for the detection of bacteria.

The results of the present study showed a significant decrease in bacterial counts in the 2.5% NaOCl group compared to the control group; in this context, almost no bacteria were detected in the 2.5% NaOCl group by the PCR technique. The bacterial counts also decreased in the photodynamic therapy group but this decrease was less than that in the 2.5% NaOCl group. The lower effect of photodynamic therapy on decreasing bacterial counts might be attributed to the fact that the existing laser probes can emit laser beam only through the tip of the probe; therefore, the beams have not reached all the straight surfaces of the root canal.

## Conclusions

Based on the results of this *in vitro* study, in which real-time PCR technique was used, 2.5% NaOCl solution resulted in a significant decrease in bacterial amounts of *E. faecalis* in contaminated root canals compared to the control group. In addition, 2.5% NaOCl was more effective than photodynamic therapy in this respect.
